# Effect of UF Membrane Rotation on Filtration Performance Using High Concentration Latex Emulsion Solution

**DOI:** 10.3390/membranes12040422

**Published:** 2022-04-14

**Authors:** Kazutaka Takata, Katsuyoshi Tanida

**Affiliations:** 1 Department of Mechanical Engineering, National Institute of Technology, Kure College, 2-2-11, Agaminami, Kure 737-8506, Japan; 2 Technical Development Group, Kobelco Eco-Solutions Co., Ltd., 1-4-78, Wakinohamacho, Chuo-ku, Kobe 651-0072, Japan; tanida.katsuyoshi@kobelco.com

**Keywords:** rotating membrane, UF membrane, latex, shear rate, boundary layer

## Abstract

A high shear rate can be applied to fluid near a membrane surface by rotating the membrane. This shear rate enables higher permeate flux and higher concentration operation when compared with a conventional cross-flow membrane since fouling and/or concentration polarization are reduced. The purpose of this study was to clarify the relationship between the fluid behavior and membrane separation characteristics of a rotating membrane surface when a latex aqueous solution was used. Due to the synergistic effect of particle removal by the centrifugal forces generated by the rotation of the membrane and the reduction in the thickness of the velocity boundary layer, membrane filtration of high-concentration slurry, which is difficult to dewater by the cross-flow method, is possible. The experimental data using an aqueous latex solution with a wide range of slurry concentrations and various membrane diameters are well correlated using a shear rate derived from the boundary layer theory. It is thus confirmed that the shear rate can be used as a design and operating parameter to define the membrane filtration characteristics.

## 1. Introduction

Membrane separation technology is widely used not only as a clarification technology for various liquids but also as a concentration technology in industrial processes. Membrane separation equipment that can create a high shear rate on the surface of the membrane reduces the concentration polarization of the membrane surface, allowing for the effective removal of solid content from the membrane surface, meaning that it can be a substitute for processes such as evaporation. This technology can therefore greatly contribute to maintaining product quality (e.g., the concentration of heat-sensitive substances). Under these circumstances, the authors here continue their research into vibratory shear enhanced processing devices and rotary membrane separation devices that can give the membrane surface a high shear rate.

With this in mind, many studies on rotating membrane systems have been conducted in the past, particularly in regions where the solution concentration is low [1] and with cylinder-type membranes that make it difficult to produce a device with a large membrane area that can be used industrially [2,3]. In recent years, different effects of rotation have been reported, such as the effect of preventing fouling by rotating the hollow fiber membrane for sewage treatment [4]. In addition, the design parameters governing the permeation characteristics of rotating cylindrical membranes have been identified [5]. In these studies, the use of different membrane rotation speeds and the shear stress applied to the fluid near the membrane surface were proposed as the parameters for evaluating the effect of membrane rotation on the membrane separation characteristics. However, the effectiveness of the proposed parameters has not been sufficiently verified. Furthermore, a device has been proposed in which an obstacle (vane) is installed between rotating membranes’ disks to disturb the fluid on the membrane surface in order to improve the separation efficiency when using low solid concentrations [6]. CFD studies have also been conducted, and back pressure in the permeate side has been clarified [7], but the parameters that govern filtration have not been verified [8]. In addition, the effect of rotation on the permeate flux in the initial stages of filtration and the effect of rotation on the rejection ratio for low-concentration fluids has been investigated by theoretical analysis, but further study is required for high concentration solutions [9]. In a comparison between a rotating membrane and a vibrating membrane, a rotating membrane with a skim milk vane and a vibrating membrane were compared, and the rotating membrane showed high permeability when using the shear rate as a parameter [10]. Furthermore, the filtration performance of beer was evaluated by a special device that simultaneously applied rotation and vibration, and it was found that the membrane separation performance was improved by the method of imparting physical movement to the membrane surface [11]. All of these examples are equipped with blades, rods, vanes, etc., between the rotating membrane disk to disturb the surface of the fluid. The separation of oil–water suspensions [12] and the evaluation of the effects of surfactants have also been investigated as applications of rotating membranes in production processes [13], and the effect of the alleviation of fouling by rotating membranes in the water purification process has also been studied [14].

As is shown in the above summary of previous studies, many applications have involved the filtration of liquids with a relatively low solid concentration. However, rotation membranes are also expected to be applied to solutions with high concentrations. Improving the rejection ratio by increasing the shear rate also enhances the industrial value of the rotating membrane, but concentrating a high-concentration solution also leads to an increase in its essential industrial value. Therefore, it is important to understand the filtration performance using a fluid with high solid concentration for the utilization of a rotating membrane system. For rotating membranes, there are many studies on the method of inserting an obstacle between the membrane discs to disturb the fluid near the membrane surface, and a basic study on the membrane separation characteristics of high-concentration solutions by a flat disc type rotating membrane is not sufficient. The parameters for evaluating the influence of operating variables such as membrane rotation speed, membrane diameter, and solution concentration on the membrane separation characteristics have not been sufficiently verified.

The present study investigates the effect of rotation on the membrane separation characteristics of a disk-type membrane under conditions where a cake layer is not formed in the membrane separation of a high-concentration solution. In this study, an ultrafiltration membrane (UF membrane) was used (material is polyether sulfone, and the molecular weight cutoff is 4000). The permeate flux was measured by changing the membrane diameter, rotation speed, and operating pressure using the latex emulsions of various solid concentrations. In addition, we investigated the parameters for the evaluation of the effect of rotation on the membrane separation characteristics of rotating membranes with various diameters from the laboratory to the commercial scale and tried to present an index for commercial scale design.

## 2. Experiment

### 2.1. Experimental Equipment

**Figure 1:**
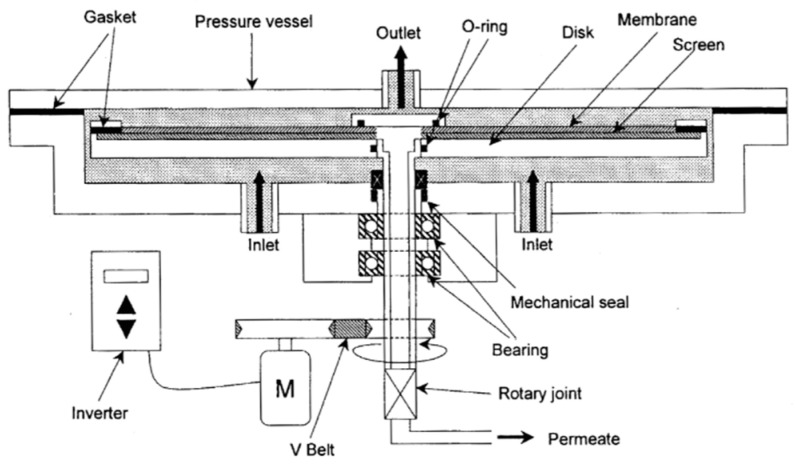
Details of rotating membrane separation apparatus.

Figure 1 shows a schematic diagram of the rotating membrane test equipment used in this study. This equipment has a structure in which a circular pressure vessel is attached to a rotating shaft, and a membrane disc with a flat membrane molded in a circle is inserted into the vessel. 

The pressure vessel is filled with the solution, and membrane separation is performed while rotating the membrane disk together with the rotating shaft.

In this experiment, this equipment was designed to be able to arbitrarily attach membrane discs with different diameters in order to conduct the experiment by changing the diameter of the membrane. The membrane disk is rotated by an electric motor connected by a rotating shaft and a saddle belt, and the rotation speed can be set to an arbitrary value by changing the frequency with an inverter. A mechanical seal is used for the rotation shaft, and the permeate water is collected in the central hole of the membrane disk (i.e., the center hole of the rotation shaft) and is discharged from the rotary joint below the rotation shaft.

The solution inlet was installed in the lower part of the pressure vessel, the outlet was installed in the upper part of the pressure vessel, and the membrane was attached to a rotating disk so that the flat membrane surface was on top. At this time, a screen was installed between the disc and the membrane to secure the permeate flow path. A gasket was used to seal the outer periphery of the disc, and a 0-ring was used to seal the inner periphery.

**Figure 2:**
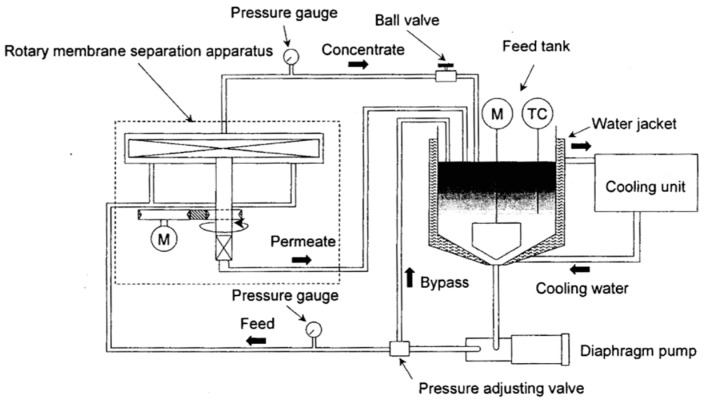
Schematic flow diagram of the experimental setup of the rotating membrane.

Figure 2 shows the flow system of the experimental apparatus used in this experiment. The equipment consists of a supply pump, a storage tank, a chiller, etc., along with the abovementioned rotating membrane device.

### 2.2. Experimental Method

In this study, the permeate flux of a latex suspension at a certain concentration for each diameter, rotation speed, and operating pressure were measured for evaluation. At this time, both the permeate water and the concentrate solution separated by the membrane were returned to the storage tank and circulated to maintain a constant concentration. The permeate flux was measured every 5 min after start rotation until the flux reached almost constant value, and the duration of each condition slightly varied, it was 10–20 min throughout whole experimental conditions. In this experiment, the latex suspension concentration, operating pressure, operating temperature, and membrane rotation speed were also measured.

### 2.3. Experimental Conditions

Table 1 shows the experimental conditions for this study. In this experiment, in order to clarify the influence of the rotation of the membrane, as shown in Table 1 (a), the experiment was conducted by changing the revolution number for the membrane to four different diameters. Here, the number of revolutions varies with the diameter of the membrane since the capacity of the motor is constant, so the upper limit of the number of revolutions decreases as the membrane disk increases up to the maximum rotation number of each disk. Four to five rotation numbers were set at almost equal intervals, so the rotation numbers were different for each disk. In addition, as shown in Table 1 (b), the operating pressure and the concentration of the latex suspension were also changed. The operating temperature and cross-flow velocity were kept to 298 K and 0.1 m/s, respectively. The kinematic viscosity of the latex emulsion is also shown in Table 1 (b).

## 3. Results and Discussion

### 3.1. Changes Transmembrane Pressure Due to Rotation

**Figure 3:**
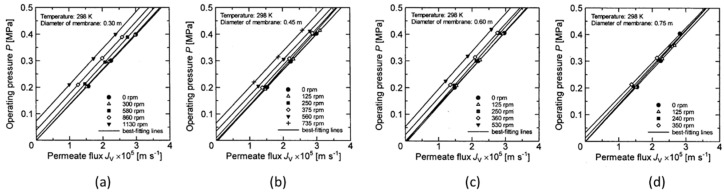
Relationship between the operating pressure and permeate water flux. (**a**) disk diameter 0.30 m (**b**) 0.45 m (**c**) 0.60 m (**d**) 0.75 m.

Matsushita et al. [15] indicated that in the separation of soy sauce cages using a rotating membrane, rotating the membrane disk causes the centrifugal force to increase the pressure on the permeate side, even when the operating pressure was constant, and therefore the permeate flux decreases due to the decrease in transmembrane pressure. Engler and Wiesner [16] also pointed out that back pressure is applied from the permeate side when the rotational speed of the membrane is large. Serra [7] also calculated back pressure in the permeate side by means of CFD. Therefore, since the membrane separation uses the transmembrane pressure as the driving force, it is necessary to calculate the accurate transmembrane pressure in consideration of this permeate pressure increase to organize the separation data of the rotating membrane. In addition, it is important to accurately grasp the increase in the pressure of the permeate since there is a risk of damage to the flat sheet membrane, especially when a reverse pressure is applied from the permeate side due to a centrifugal force. However, previous studies have only pointed out permeate pressure increases without examining the magnitude of the pressure increase or the parameters that affect the permeate flux. In this study, a membrane separation experiment using a latex suspension was performed by changing the diameter, rotation speed of the membrane disk, and operating pressure. Therefore, it is necessary to accurately grasp the pressure increase on the permeate side when comparing the filtration performance based on the transmembrane pressure when the membrane disk diameter or rotation speed changes. Firstly, the permeate flux of pure water was measured in order to investigate the effect of the permeate pressure increase caused by the rotation of the membrane disk on the permeate flux. The results are shown in Figure 3a–d for each membrane disc diameter. From these figures, we can see that the operating pressure and the permeate flux of pure water are in a linear proportional relationship at any membrane diameter and number of revolutions, and the operating pressure rises with increasing rotation speed even when the permeate flux is constant. It is found that the slopes of these lines are almost constant at any membrane diameter and rotation speed and that only the intercept value changes. The increase in permeate pressure due to the rotation of the membrane has a specific value for a particular combination of membrane diameter and rotation speed. In other words, the values of the intercepts of the lines shown in Figure 3 are considered to be the pressure increase of the permeate at each rotational speed of the disc used in this experiment. Therefore, the relationship between the rotation of the membrane and the increase in permeate pressure was examined as follows.

**Figure 4:**
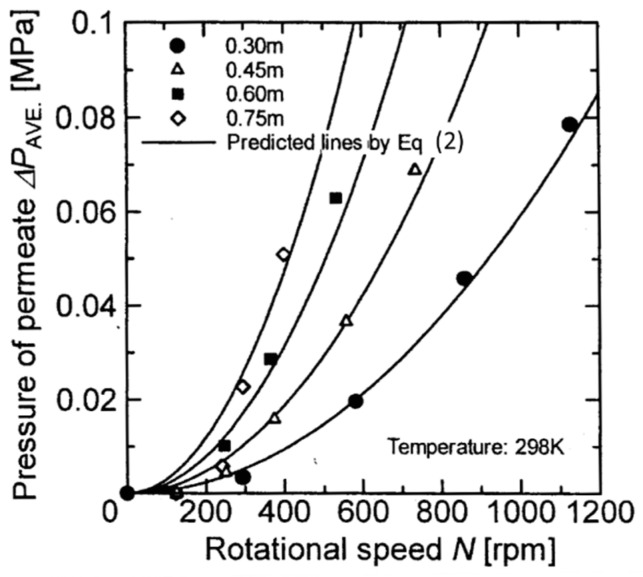
Relationship between permeate pressure and rotation speed.

The pressure difference between two points given by the centrifugal force acting on the rotating object is expressed by the following equation:

(1) $$∆P=\frac{\rho \omega^{2}}{2}\left(r_{2}^{2}-r_{1}^{2}\right)$$

where Δ*P* [Pa] is the pressure difference between two points given by the centrifugal force of rotation, *r*_1_ [m] and *r*_2_ [m] are the inner and outer radius, respectively, *ρ* [kg/m^3^] is the density, and *ω* [rad/s] is the angular velocity of the rotating obstacle. The rotating obstacle here is the permeate water. Since Equation (1) represents the pressure difference between the two rotating points, the pressure applied to the permeate on the outer periphery of the membrane disk is obtained using *r*_1_ as the inner radius of the membrane, *r*_2_ as the outer radius of the membrane, and ρ as the permeate density. However, the permeate pressure increment value obtained in the experiment is the value obtained from the relationship between the operating pressure and the permeate side, and it is therefore the average permeate pressure increment value across the membrane disk. In other words, in order to compare Equation (1) with the permeate pressure increment obtained in this experiment, it is necessary to calculate the average value by integrating the radial direction. Thus, Equation (1) is integrated in the radial direction and the average value of the pressure increment applied to the permeate by the centrifugal force due to the rotation of the membrane is obtained. The result is shown in the following equation:

(2) $$∆P_{AVE}=\frac{\rho \omega^{2}}{6}\frac{r_{2}^{3}-r_{1}^{3}}{r_{2}-r_{1}}$$

where Δ*P_AVE_* [Pa] is the average value of the pressure increment on the permeate side caused by the centrifugal force of the rotation. Figure 4 shows the comparisons of the permeate pressure increment obtained from Equation (2) with the permeate pressure increment obtained from the intercept value in Figure 3. From this figure, it is found that the estimated value of Equation (2) agrees well with the experimental data. This result shows that the change in the relationship between the operation pressure of pure water and the permeate flux due to the rotation of the membrane shown in Figure 3 occurs since the centrifugal force generated by the rotation of the membrane increases the pressure of the permeate. This result shows that the increment of permeate side pressure by the centrifugal force due to rotation shown in Figure 3 causes the change in the relationship between the operating pressure and the permeate flux due to the rotation of the membrane.

Although this result is for pure water, the pressure increase on the permeate side due to the rotation is considered to be the same in the experiments using latex solutions. This is due to the fact that, in this study, a membrane with a low molecular weight cut-off is used, and neither latex particles nor dispersant pass through the membrane, so the permeate water has almost the same physical properties as pure water.

When considering the rigor of the experimental results, it is necessary to measure the pressure increment of the permeate side using a latex solution. However, in the case of a latex solution, the permeate flux increases when the operating pressure is increased, but at the same time the latex concentration on the membrane surface increases due to concentration polarization, resulting in permeation resistance. Therefore, the linear relationship between the permeate flux of the latex solution and the operating pressure as shown in Figure 3 is not obtained. Thus, in the experiment using a latex solution, the transmembrane pressure was evaluated by subtracting the experimental value shown in Figure 4 as the pressure increase value of the permeate from the operating pressure.

### 3.2. Relationship between Transmembrane Pressure and Permeate Flux

**Figure 5:**
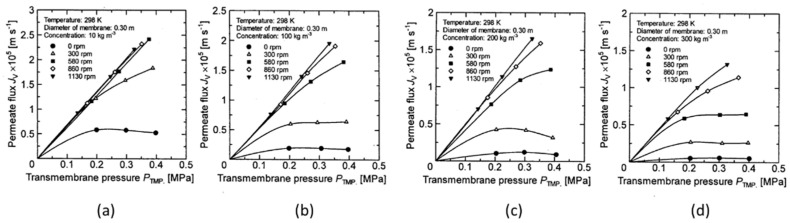
Relationship between measured TMP and permeate flux for disk diameter 0.3 m. (**a**) at latex concentration 10 kg/m^3^ (**b**) 100 kg/m^3^ (**c**) 200 kg/m^3^ (**d**) 300 kg/m^3^.

**Figure 6:**
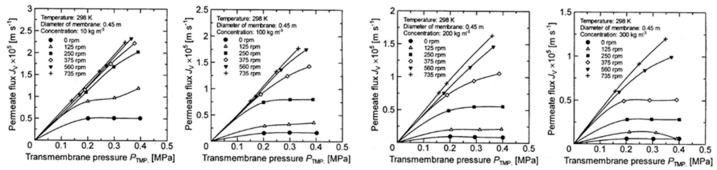
Relationship between measured TMP and permeate flux for disk diameter 0.45 m. (**a**) at latex concentration 10 kg/m^3^ (**b**) 100 kg/m^3^ (**c**) 200 kg/m^3^ (**d**) 300 kg/m^3^.

**Figure 7:**
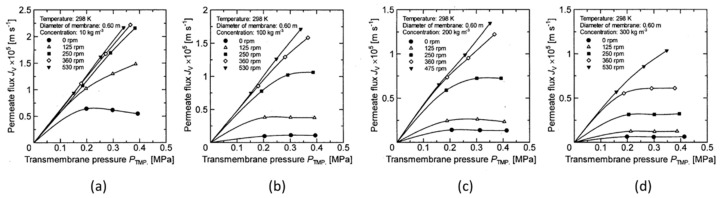
Relationship between measured TMP and permeate flux for disk diameter 0.6 m. (**a**) at latex concentration 10 kg/m^3^ (**b**) 100 kg/m^3^ (**c**) 200 kg/m^3^ (**d**) 300 kg/m^3^.

**Figure 8:**
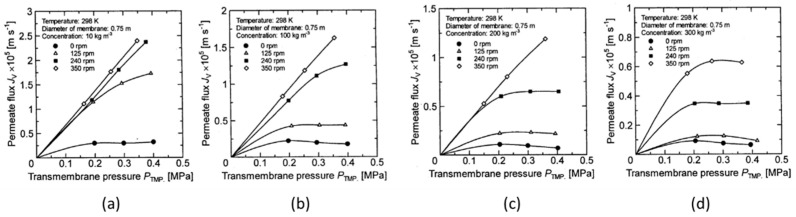
Relationship between measured TMP and permeate flux for disk diameter 0.75 m. (**a**) at latex concentration 10 kg/m^3^ (**b**) 100 kg/m^3^ (**c**) 200 kg/m^3^ (**d**) 300 kg/m^3^.

Figure 5, Figure 6, Figure 7 and Figure 8 show the relationship between the measured permeate flux and the trans membrane pressure in the experiments using a latex solution under the experimental conditions shown in Table 1.

It is found that the permeate flux increases with increasing membrane rotation, and the rate of increase in permeate flux with respect to the transmembrane pressure increases. At a low concentration, the relationship between the permeate flux and the transmembrane pressure becomes linear at a certain rotation, and the value is almost the same (for example, it is above 580 rpm in Figure 5a).

**Figure 9:**
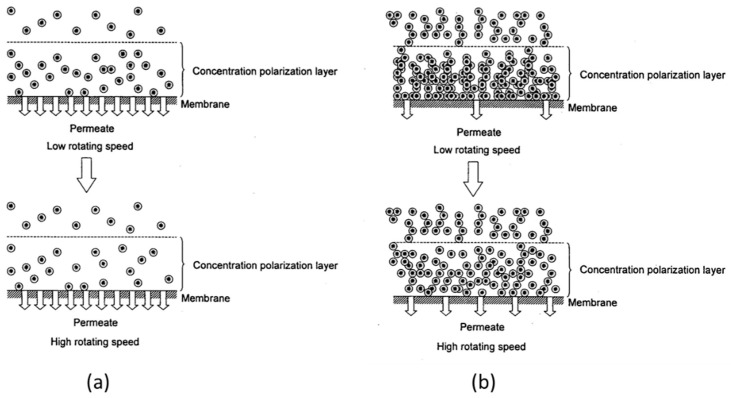
Schematics of concentration polarization layer of rotating membrane surface. (**a**) low concentration (**b**) high concentration.

However, as the concentration increases, the rotation number at which the linear relationship between the permeate flux and the transmembrane pressure begins increases (for example, 860 rpm in Figure 6b and 1130 rpm in Figure 6c). Furthermore, no linear relationship can be seen at any rotation number at a concentration of 300 kg/m^3^. The effect of the rotation number on the relationship between the permeate flux and the transmembrane pressure is explained as follows using the schematic diagram of the concentration polarization shown in Figure 9.

In the membrane separation of a solution containing a solute, a solute that is close to the membrane surface due to a concentration polarization phenomenon develops permeation resistance. Therefore, the permeate flux is greatly affected by the solute concentration near the membrane surface. For example, the lower the solute concentration near the membrane surface, the larger the permeate flux. 

In a rotating membrane separation system, the reduction effect of concentration polarization occurs in which the concentration near the membrane surface decreases due to the mixing effect between the membrane surface and the solution caused by the rotation. When the number of rotations of the membrane increases, the solute concentration near the membrane surface decreases and the permeate flux increases.

However, when the concentration of the solute is low, as shown in Figure 9a, the increase in the solute concentration near the film surface due to the concentration polarization phenomenon is small, as shown in the upper diagram of Figure 9a, so the reduction effect of concentration polarization reaches a constant value when the rotation speed reaches a certain number. Additionally, even if the rotation number is increased further, the concentration near the membrane surface does not decrease, as shown in Figure 9a, so the permeate flux is considered to be almost a constant value. On the other hand, in the case of a high concentration, as shown in Figure 9b, the increase in the concentration near the membrane surface due to the concentration polarization is large, as is shown in the upper diagram of Figure 9b. The effect of reducing the concentration polarization due to rotation does not reach the saturation value in the range of rotation numbers in this study. Therefore, if the rotation number is increased, as shown in the lower diagram of Figure 9b, the concentration near the membrane surface decreases, and the dependence of the permeate flux on the rotational speed appears in the high rotational speed range. These results suggest that the effect of the rotation of the membrane on the permeate flux is particularly large at high concentrations.

### 3.3. Relationship between Rotation Number and Permeate Flux

**Figure 10:**
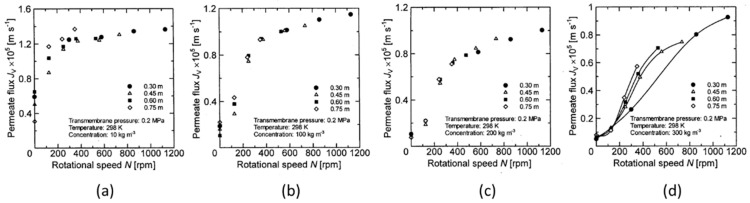
Relationship between rotation speed and permeate flux when the TMP is 0.2 Mpa. (**a**) at latex concentration 10 kg/m^3^ (**b**) 100 kg/m^3^ (**c**) 200 kg/m^3^ (**d**) 300 kg/m^3^.

**Figure 11:**
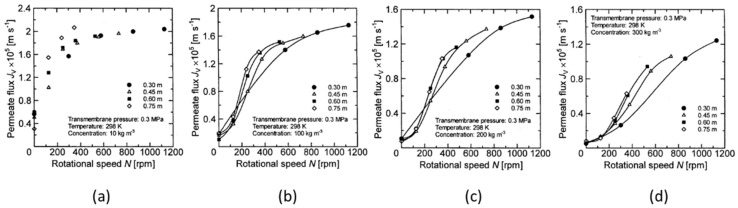
Relationship between rotation speed and permeate flux when the TMP is 0.3 Mpa. (**a**) at latex concentration 10 kg/m^3^ (**b**) 100 kg/m^3^ (**c**) 200 kg/m^3^ (**d**) 300 kg/m^3^.

From the results in the previous section, it is found that the permeate flux of the latex suspension was changed by the rotation, even when the transmembrane pressure was the same. Therefore, there likely exists a parameter that makes it possible to quantitatively evaluate the influence of rotation on the membrane separation characteristics. First, the relationship between the rotation number and the permeate flux is clarified to determine whether the rotation number is applied as an evaluation parameter. The relationship between membrane rotation number and permeate flux at 0.2 MPa and 0.3 MPa trans membrane pressure were studied. The results are shown in Figure 10 and Figure 11.

Here, the solid line in the figure shows a plot with only a clear difference in permeate flux for each membrane disk. From these figures, it can be seen that the permeate flux of the latex suspension increases with increasing membrane rotation speed. In particular, when the latex concentration is low (e.g., concentration conditions of 10 kg/m^3^, as shown in Figure 10a and Figure 11a), the permeate flux approaches a certain value in the high rotation number region. It can be seen that the permeate flux does not increase, even if the rotation number is further increased (in Figure 10a, about 300 rpm or more). On the other hand, the permeate flux continues to increase with the rotation number in other experimental conditions. As described in the previous section, when the latex concentration is low, the increase in latex concentration near the membrane surface due to the concentration polarization phenomenon is small. Therefore, the permeate flux does not increase since the effect of reducing the concentration polarization due to the rotation of the membrane is small, and the concentration near the membrane surface does not decrease even if the rotation speed is increased. Additionally, Figure 10a–c and Figure 11a show that there is little change in the permeate flux with respect to the membrane rotation speed even when the membrane diameter changes, although it varies slightly in the low rotation number range. On the other hand, it is found from Figure 10d and Figure 11b–d that the change in the permeate flux with respect to the membrane rotation speed differs depending on the membrane diameter, and the permeate flux increases when increasing the membrane diameter. This means that the decreasing effect of the concentration polarization is not affected by the rotation when the increase in the solid concentration near the membrane surface is due to the concentration polarization phenomenon with a small solid concentration or small transmembrane pressure, even if the membrane diameter changes.

On the other hand, under conditions where there is a large increase in the solid concentration near the membrane surface due to concentration polarization, such as high concentration and high transmembrane pressure, the change in the membrane diameter has a significant effect on the reduction effect on the concentration polarization due to membrane rotation. As described above, the relationship between the rotation number and the permeate flux is the same, even when the membrane diameter changes under low concentration and low transmembrane pressure, and therefore, it was found that the number of rotations can be used as a parameter to evaluate the effect of the rotation number on membrane separation characteristics under the aforementioned conditions. However, as the relationship between the rotation number and the permeate flux varies with the membrane diameter under high concentrations and high transmembrane pressures, the effect of membrane rotation on the membrane separation characteristics cannot be fully explained using only membrane rotation number as a parameter. From the above results, it is necessary to evaluate the change in membrane diameter together with the rotation number as a parameter that affects the influence on membrane separation characteristics.

### 3.4. Relation between Membrane Rotation Velocity and Permeate Flux

**Figure 12:**
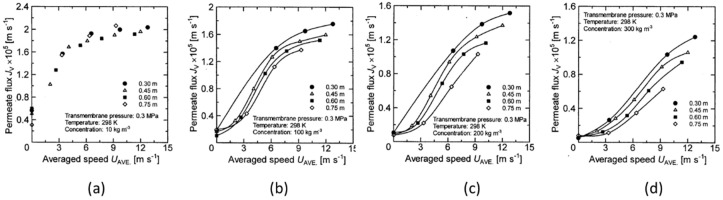
Relationship between average speed and permeate flux when the TMP is 0.2 Mpa. (**a**) at latex concentration 10 kg/m^3^ (**b**) 100 kg/m^3^ (**c**) 200 kg/m^3^ (**d**) 300 kg/m^3^.

**Figure 13:**
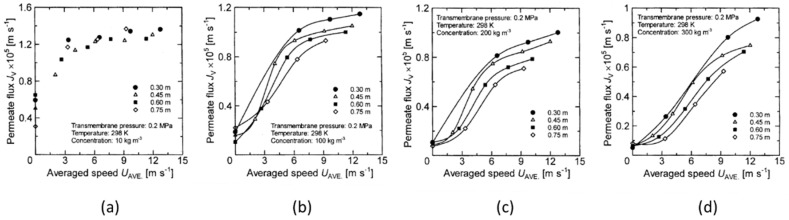
Relationship between average speed and permeate flux when the TMP is 0.3 Mpa. (**a**) at latex concentration 10 kg/m^3^ (**b**) 100 kg/m^3^ (**c**) 200 kg/m^3^ (**d**) 300 kg/m^3^.

From the results obtained from the previous section, it was shown that apart from the membrane rotation number, the influence of the membrane diameter should also be considered as a parameter for evaluating the influence of membrane rotation on membrane separation characteristics. Here, the membrane rotation velocity is defined as the product of the membrane diameter and the rotation number, and the relationship between the membrane rotation velocity and the permeate flux is shown in Figure 12 and Figure 13 in order to examine whether the parameter is valid as an evaluation factor. Here, since the membrane rotation velocity varies with the diameter of the membrane disk, the average membrane rotation velocity expressed by the following equation was used as a parameter:

(3) $$U_{AVE}=\frac{2\omega }{3}\frac{r_{2}^{3}-r_{1}^{3}}{r_{2}^{2}-r_{1}^{2}}$$

where *U_AVE_* [m/s] is the average membrane rotation velocity of the disk. Figure 12 and Figure 13 show the effect of *U_AVE_* on the permeate flux at a transmembrane pressure of 0.2 MPa and 0.3 MPa over a wide range of latex concentrations. Here, as in the case of Figure 10 and Figure 11, the solid line represents the data for which the influence of rotation number on the permeate flux can be seen for each membrane disk. From these figures, it can be seen that permeate flux increases when increasing the average membrane rotation velocity, as was the case when rotation number was a parameter. Further, comparing the results of Figure 12a and Figure 13a where the latex concentration is 10 kg/m^3^, the variation in the permeate flux with respect to the change in the membrane disk diameter is slightly larger. As shown in Figure 10a and Figure 11a, it shows the same tendency as the effect of the rotation number on permeate flux. However, it is found that the permeate flux changes with changes in the membrane disk diameter, even under low latex concentrations. This is different from the change in permeate flux with respect to membrane rotation number. Further, as the latex concentration increases, the permeate flux decreases as the membrane disk diameter increases. This is thought to be due to the effect of the membrane diameter being taken into account by using average membrane rotation velocity as a parameter at high concentrations. However, when the average membrane rotation velocity is used as a parameter, the permeate flux depends on the membrane diameter more than when the membrane rotation number is used as a parameter. Therefore, the averaged membrane rotation velocity is also considered to be insufficient to accurately represent the effect of membrane rotation on the membrane separation characteristics.

### 3.5. Relationship between Share Rate and Permeate Flux

**Figure 14:**
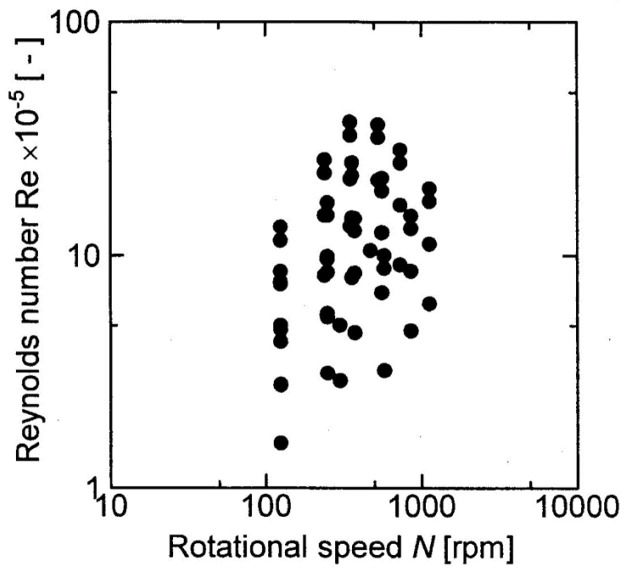
Plot of Reynolds number against rotational speed.

From the results of previous sections, it was found that the effect of rotation on the membrane separation characteristics could not be expressed sufficiently, even if the membrane rotation number and membrane rotation velocity were used as parameters. Therefore, we examine whether the shear rate of the membrane surface is effective as a parameter for representing the filtration performance. Generally, the shear rate applied to the fluid near the membrane surface is given by the velocity scale divided by the length scale and the rotation velocity of the membrane is also used for the rotating membrane. For the length scale, the thickness of the velocity boundary layer formed on the rotating disk is used. First, the rotational velocity at any position of the membrane used as the velocity scale is given by the following equation as the product of the membrane radius and the angular velocity of rotation:

(4) U=rω

where *U* [m/s] is the rotation velocity at a velocity scale and *r* [m] is the radius at any position of the membrane. The equation representing the thickness of the velocity boundary layer formed on the rotating disk varies depending on whether the flow on the rotating disk is laminar or turbulent. Therefore, the Reynolds number of the fluid on the rotating disk was calculated under the rotating conditions in this experiment, and this determined whether the flow was laminar or turbulent. The Reynolds number of the fluid on the rotating disk is expressed by the following equation:

(5) $$Re=\frac{r_{2}^{2}\omega }{\nu }$$

where *Re* [-] is the Reynolds number, *ν* [m^2^/s] is the kinematic viscosity of the latex suspension, and the values shown in Table 1 (b) were used for evaluation. Figure 14 shows the calculated Reynolds number of the fluid on the rotating disk in this experiment. According to this figure, the Reynolds number of the fluid on the rotating disk under these experimental conditions is of the order of 10^5^–10^6^, and the flow of the fluid on the rotating disk was in the range of transition to turbulence [17]. Therefore, in this study, the velocity boundary layer thickness in the turbulent region was used as the thickness of the velocity boundary layer formed on the rotating disk. The thickness of the velocity boundary layer in the case of turbulent flow is given by the following equation [18]:

(6) $$\delta_{v}=0.526\dot{\gamma }{\left(\frac{\nu }{\omega r^{2}}\right)}^{1/5}$$

where *δv* [m] is the thickness of the boundary layer. Therefore, the shear rate $\dot{\gamma }$ [1/s] given to the fluid near the membrane surface by the rotation of the membrane under this experimental condition is expressed by dividing Equation (4) by Equation (6).

(7) $$\dot{\gamma }=\frac{U}{\delta_{v}}=\frac{r^{0.4}\omega^{1.2}}{0.526\nu^{0.2}}$$

Equation (7) shows that the shear rate of the rotating membrane is proportional to the radius’ 0.4th power, the angular velocity’s 1.2th power, and the fluid kinematic viscosity’s −0.2th power. As is clear from Equations (4), (5) and (7), the membrane used in this experiment has a donut shape, so the rotational velocity and boundary layer thickness change in the radial direction of the membrane. Therefore, the shear rate applied to the fluid near the membrane surface also changes in the radial direction. Therefore, it is necessary to evaluate membrane separation performance using the average value of the shear rate given to the entire membrane surface. The average shear rate is expressed by the following equation by integrating Equation (7) in the radial direction and dividing by the membrane area:

(8) $${\dot{\gamma }}_{AVE}=\frac{\omega^{1.2}}{0.631\nu^{0.2}}\frac{r_{2}^{2.4}-r_{1}^{2.4}}{r_{2}^{2}-r_{1}^{2}}$$

**Figure 15:**
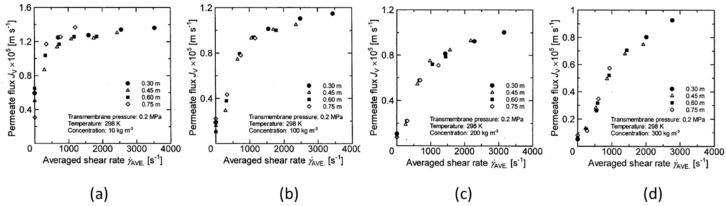
Relationship between average shear-rate and permeate flux when the TMP is 0.2 Mpa. (**a**) at latex concentration 10 kg/m^3^ (**b**) 100 kg/m^3^ (**c**) 200 kg/m^3^ (**d**) 300 kg/m^3^.

**Figure 16:**
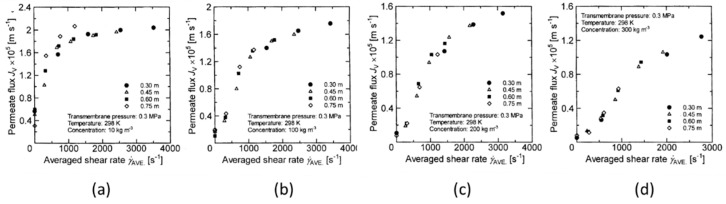
Relationship between average shear-rate and permeate flux when the TMP is 0.3 Mpa. (**a**) at latex concentration 10 kg/m^3^ (**b**) 100 kg/m^3^ (**c**) 200 kg/m^3^ (**d**) 300 kg/m^3^.

In order to examine the validity of the average shear rate as an evaluation parameter, the relationship between the average shear rate and the permeate flux at the transmembrane pressures of 0.2 MPa and 0.3 MPa was obtained. Figure 15 and Figure 16 show that the permeate flux increases as the average shear rate increases.

Unlike when using the membrane rotation velocity and the membrane rotation number, the fitting is well performed without much variation in the permeate flux with respect to the change in the membrane diameter. This indicates that by using the shear rate and considering the velocity boundary layer as a parameter, the effect of membrane rotation on the membrane separation characteristics can be expressed more accurately than in the case of using membrane rotation number or membrane rotational velocity.

From the above results, it was clarified that the shear rate derived from the thickness of the velocity boundary layer considering the rotation of the membrane has a great influence on the membrane separation characteristics of the rotating membrane.

### 3.6. Index for Scale-Up Procedure

Here, we indicate the outline of a design index toward the large-scale utilization of the obtained permeate flux with different disk diameters and concentrations. In the experiments, the flow rate was determined to maintain a 0.1 m/s average flow velocity in each disk diameter. Here, the supplying fluid is assumed to flow between each disk and to determine the diameter of large equipment when calculating the average shear rate. In addition, since the permeate flux is proportional to the shear rate regardless of the membrane diameter, the relationship between the permeate flux and the shear rate at each concentration is obtained from the experimental data of the small equipment. The average shear rate of large equipment is applied to this relationship to determine the permeate flux corresponding to the shear rate of the large equipment at each concentration. Using this relationship, the permeate flux of each disk is calculated from the concentration of the fluid at the inlet of the disk and this is used to determine the concentration and flow rate of the fluid at the next disk. This calculation is repeated until the last disk. As a result of this calculation, there is a concentration gradient from the inlet to the outlet of the equipment, and the permeate flux decreases as the fluid moved towards the outlet of the membrane module. If the outlet concentration does not reach the design concentration, it is necessary to process the fluid using a circulation operation or to include second stage equipment.

As described above, the idea of a design index with different disk diameters was presented, and these types of methods are made possible by the correlation of the permeate flux with the shear rate. The degree of freedom of the design increases when the membrane disks are segmented.

## 4. Conclusions

In this study, a membrane separation experiment involving a latex suspension was carried out by changing the membrane diameter and operating conditions of the rotating membrane, and the effect of the membrane rotation on the membrane separation characteristics was clarified. In addition, the parameters determining the effect of rotation on the membrane separation characteristics were discussed. The results are summarized as follows.

1. The relationship between the operating pressure and the pure water permeate flux changes when rotating the membrane. This is due to the fact that the pressure of the permeate side increases due to the centrifugal force acting on the rotation of the membrane. This was confirmed by the good agreement between the experimental value and the value obtained from the equation representing the pressure difference generated by the action of centrifugal forces between any two points on the rotating disk.
2. The permeate flux of the rotating membrane is almost constant at certain rotation numbers at low latex concentrations but continues to rise at high concentrations. This is due to the fact that the higher the concentration, the greater the effect of membrane rotation on the permeate flux. Under any operating condition and membrane diameter, the permeate flux of the latex solution increases when increasing the membrane rotation number and rotation velocity. However, the relationship between the rotation parameter and the permeate flux changes when the membrane diameter changes. This suggests that neither the rotation number nor the rotation velocity of the membrane is a parameter that accurately represents the influence of membrane rotation on the membrane separation characteristics.
3. The permeate flux of the latex solution increases when increasing the average shear rate applied to the fluid near the membrane surface under all operating conditions. Furthermore, since the relationship between the average shear rate and the permeate flux does not change even when the membrane diameter changes, the average shear rate is a parameter that accurately represents the effect of membrane rotation on the permeate flux.

## Figures and Tables

**Table 1:** Experimental conditions.

(**a**) Rotating conditions for rotary membrane separation experiments.			
**Membrane Diameter** **(m)**		**Rotation Speed** **(rpm)**	
0.30		0, 300, 580, 860, 1130	
0.45		0, 125, 250, 375, 560, 735	
0.60		0, 125, 250, 360, 475, 530	
0.70		0, 125, 240, 350	
(**b**) Operating conditions.			
**Operating Pressure** **(MPa)**	**Concentration of Latex Emulsion** **(kg m^−3^)**		**Kinematic Viscosity of Latex Emulsion** **(m^2^ s^−1^)**
0.2	10		1.365 × 10^−6^
0.3	100		1.546 × 10^−6^
0.4	200		2.329 × 10^−6^
	300		4.237 × 10^−6^

## Data Availability

Not applicable.
